# Generating hepatitis B and D monitoring indicators in Germany using claims data: number of persons tested, incident and prevalent infections, 2016–2021

**DOI:** 10.1186/s12879-026-13706-8

**Published:** 2026-06-02

**Authors:** Lisa Branke, Alexandra Hofmann, Anastassiya Stepanovich-Falke, Marco Alibone, Viviane Bremer, Ruth Zimmermann, Sandra Dudareva

**Affiliations:** 1 Department of Infectious Disease Epidemiology, Robert Koch Institute, Berlin, Germany; 2 Charité – Universitätsmedizin Berlin, Corporate Member of Freie Universität Berlin and Humboldt-Universität zu Berlin, Berlin, Germany; 3 InGef - Institute for Applied Health Research Berlin GmbH [Institut für angewandte Gesundheitsforschung Berlin GmbH], Berlin, Germany; 4 Institute of Public Health, Riga Stradins University, Riga, Latvia

**Keywords:** Claims data, Germany, Hepatitis B, Hepatitis D, Incidence, Indicators, Prevalence, Testing

## Abstract

**Background:**

Hepatitis B virus (HBV) infection remains one of the most common infectious diseases globally and can be exacerbated by coinfection with hepatitis D virus (HDV). In Germany, the incidence of hepatitis is reported annually through mandatory notification data. Nevertheless, these data lack information on hepatitis prevalence and testing coverage. We evaluated whether statutory health insurance claims data are suitable for continuous monitoring of key hepatitis indicators and whether they can complement existing HBV and HDV surveillance with reliable, population-based estimates.

**Methods:**

We conducted a population-based cohort study using anonymised claims data from an age- and gender-representative sample from the InGef (Institute for Applied Health research) database between 2016 and 2021. We retrospectively analysed monitoring indicators for HBV and HDV -including annual prevalent infections within the respective analysis year, incidence of newly detected HBV infections (defined using a 3-year diagnosis-free interval) and the number of persons tested- based on coded diagnoses among individuals with access to healthcare services. Results were extrapolated to the German total population.

**Results:**

Between 1,996 and 2,066 persons per 100,000 population were tested annually for at least one HBV marker (HBsAg or Anti-HBc), with 97% undergoing HBsAg testing only. The proportion of annual prevalent HBV infections ranged from 0.14% to 0.15% between 2016 and 2020. Incidence of newly detected HBV infections (3-year diagnosis-free interval) declined from 13.5 per 100,000 in 2019 to 8.9 per 100,000 in 2020. The proportion of prevalent HDV infections among HBV-positive persons varied from 4.5 to 6.4%.

**Conclusions:**

Claims data provide a viable basis for continuous monitoring of selected hepatitis indicators in Germany. The number of prevalent and incident infections is consistent with other data sources and we were able to estimate the number of persons tested for hepatitis, addressing an important data gap. Claims data complement current surveillance systems and provide information for targeted public health action. The established approach should be applied to generate further hepatitis monitoring indicators.

**Trial registration:**

Clinical trial number: Not applicable.

**Supplementary Information:**

The online version contains supplementary material available at 10.1186/s12879-026-13706-8.

## Background

Hepatitis B virus (HBV) infection is one of the most common infectious diseases, chronic infection affecting more than 250 million people worldwide [1]. A chronic HBV infection increases the risk for liver diseases, such as cirrhosis and hepatocellular carcinoma (HCC). In 2022, the World Health Organization (WHO) estimated 1.1 million deaths, mostly resulting from aforementioned comorbidities [1]. Persons living with HBV can coinfect with Hepatitis D virus (HDV), as HDV requires HBV for its replication. HDV infection is considered the most severe form of viral hepatitis, due to its association with a faster progression to liver-related death and HCC. Globally, approximately 5% of persons living with HBV are coinfected with HDV [2].

In 2016, WHO implemented a strategy to eliminate viral hepatitis as a public health threat until 2030. This strategy provides a framework for assessing the health situation and trends in order to improve the national health sector response [3, 4]. The strategy comprises indicators for monitoring, that are divided into core indicators, such as “*prevalence of chronic HBV infection” (C.1a)* and additional indicators, such as “*Hepatitis D coinfection among people living with chronic HBV infection” (A.1)* and *“Hepatitis B testing” (A.5).*

Although nearly a decade has passed since the WHO defined hepatitis elimination indicators [3, 5], many countries, including Germany, continue to face substantial data gaps for comprehensive hepatitis surveillance [6–8]. In Germany, the incidence of hepatitis is reported annually through mandatory notification data according to the Infection Protection Act and is comparatively low. However, these data lack key epidemiological dimensions, particularly with regard to testing coverage and broader information on diagnosed hepatitis infections in the population, which limits comprehensive assessment of progress toward hepatitis elimination goals. The last population-based seroprevalence study on hepatitis B and C was conducted in 2011 [9, 10], reporting a low prevalence of 0.3% in the general population. Since then, no updated seroprevalence data have been collected and the number of individuals tested for HBV remains unknown.

In Germany, access to centralized registries is regulated and existing surveillance systems currently provide limited information beyond notified cases. The establishment of new data collection systems is feasible but requires considerable resources. As reliable and continuous monitoring of selected hepatitis indicators requires broader data inputs than given in notification data, alternative information systems are needed to expand available epidemiological information. Routinely collected statutory health insurance claims data may represent a valuable complementary data source, as approximately 85% of the German population is covered by statutory insurance [11]. Claims data contain records of outpatient and inpatient services, diagnostic codes and laboratory testing, enabling regular estimation of healthcare utilisation and disease burden in an age- and sex-representative sample.

In this study, we examined whether claims data can be used as additional data source for viral hepatitis monitoring indicator generation. From claims data we generated information on selected population-level monitoring indicators: annual number of persons tested for HBV and HDV, prevalent and newly detected HBV infections as well as prevalent HDV infections among HBV-positive persons. While linkage to care is an important component of the hepatitis care cascade, it cannot be directly assessed using claims data due to the lack of detailed clinical pathway information. Based on results, we discuss suitability of claims data focusing on plausibility of observed trends and to what extent the results are consistent with existing national data sources. Additionally, we assessed if the established analysis can be used to report on respective WHO hepatitis monitoring indicators.

## Methods

### Database and ethics

The study is based on an age- and gender-representative sample of 4 million statutorily insured individuals in Germany (approximately 5% of the German population), drawn from the research database of InGef (Institute for Applied Health Research Berlin GmbH) [11]. The database contains anonymised routine data, mainly from Company Health Insurance Funds and Craft Guild Health Insurances (Betriebs- und Innungskrankenkassen). Company Health Insurances were originally established by larger employers to provide health coverage for their employees, whereas Craft Guild Health Insurances historically covered workers in specific skilled trades. Today, both types of funds are open to members beyond their original occupational groups.

For the study, we used both individual and demographic characteristics such as age, gender, and insurance periods, as well as data from outpatient and inpatient care, including diagnoses coded according to the 10th International Classification of Diseases in the German Modification (ICD-10 GM) and examinations carried out by physicians based on the doctor’s fee scale (Einheitlicher Bewertungsmaßstab, EBM). In Germany, outpatient services are reimbursed based on fee schedule items (Gebührenordnungsposition, GOP) from the EBM, which assign monetary values to medical services.

The InGef research database contains fully anonymised routine healthcare claims data that fulfil all applicable requirements of German data protection law. According to current German regulations and guidelines for secondary data analysis, studies based exclusively on anonymised secondary data do not require approval by an institutional review board or ethics committee [12, 13].

### Study design and outcomes

We conducted a population-based retrospective cohort study. We cross-sectionally analysed several outcomes within a representative sample between 2016 and 2021 and extrapolated these to the German total population: (1) annual number of persons tested for HBV and HDV, (2) annual proportion of prevalent HBV infections, (3) annual prevalent HDV infections among HBV-positives and (4) incidence of newly detected HBV infections. These monitoring indicators are derived at the population level and are not intended for individual patient monitoring. Rather, claims data are used as an additional data source to complement existing surveillance systems. Because claims data reflect only diagnosed and coded persons among individuals with access to healthcare services, uninsured and/or undiagnosed persons are not represented in these prevalence and incidence estimates.

Prevalent infections and incidence of newly detected HBV infections were determined using a standardised definition: individuals were classified as having a HBV infection if they had at least one inpatient diagnosis or at least two confirmed outpatient diagnoses recorded in different quarters (so-called M2Q criterion) within a 12-month period. This approach minimizes the risk of misclassification due to coding errors in outpatient settings. The number of persons tested was identified using GOPs in outpatient settings. To determine the appropriate diagnostic codes with regard to potential coding changes over time, we viewed different versions of EBM catalogues [14].

**Fig. 1:**
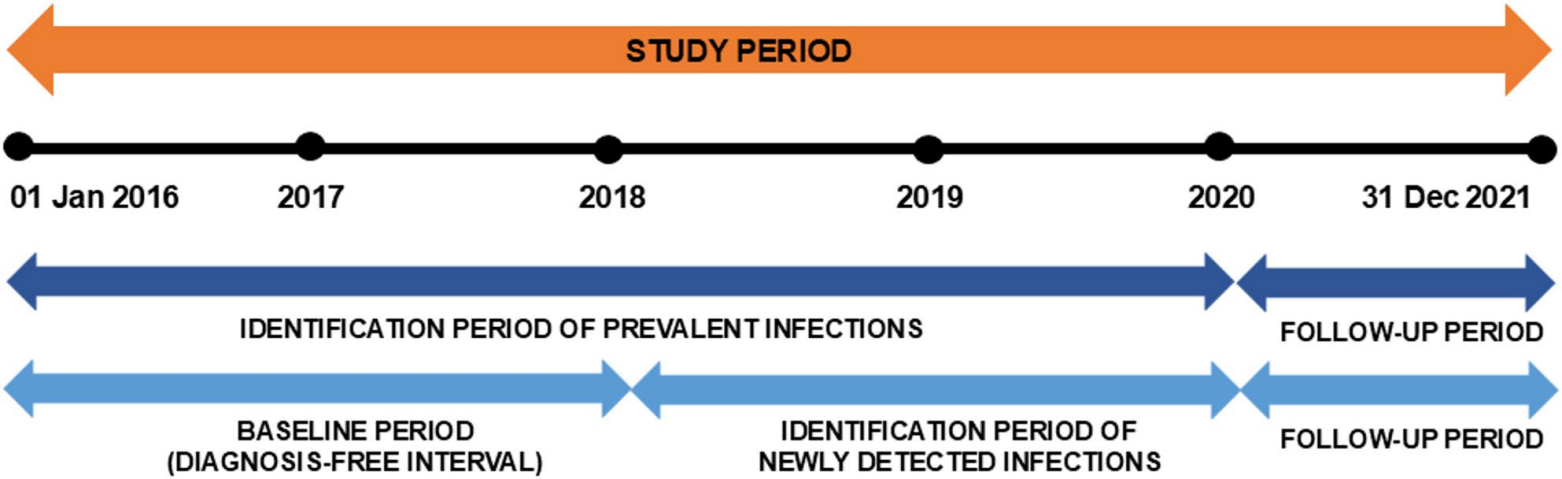
Diagram indicating time periods for analysing monitoring indicators

The first quarter in which an ICD diagnosis or GOP for HBV/HDV testing appeared was defined the index quarter. For cross-year validation of outpatient diagnoses, individuals had to be observable during the subsequent year (follow-up period). In patients where diagnoses occurred over multiple years, the infection was attributed to the year of the first qualifying diagnosis (index year). To identify newly detected infections, a 3-year diagnosis-free interval (baseline period) was used (see Fig. 1).

### Study population

The study population included individuals from the InGef analysis sample, who were residents of Germany as of 1 January of the respective analysis year (2016–2021). Furthermore, all persons had to be continuously insured during the analysis year. For validation of outpatient diagnosis according to the M2Q criterion, continuous insurance in the subsequent year was also required. To determine the incidence of newly detected HBV infections (3-year diagnosis-free interval), only persons who were still at risk of being newly identified with a HBV infection at the time of cohort entry (on 1 January of each year) were included. Persons who already had a prevalent HBV diagnosis within the 3-year baseline period and persons who were not continuously observable during this period were therefore excluded.

### Definitions

#### Persons tested for HBV

The identification of persons tested was based on laboratory diagnostic markers. We devided persons tested for HBV in two groups: first, persons with at least one HBsAg or Anti-HBc test (≥ 1 marker), understood as initial testing with regard to the national guideline [15]. Second, persons who were tested for at least two of the following markers (≥ 2 markers) within the same quarter, regardless to combination: (HBsAg **OR** Anti-HBc) **AND** (DNA **OR** HBeAg **OR** Anti-HBc **OR** Anti-HBc IgM **OR** Anti-HBe). For the description of the used GOPs and their combination for fulfilling ≥ 1 marker or ≥ 2 markers criterion see Tables 1 and 2.

**Table 1:** GOPs and their description in accordance to the doctors’ fee scale

Virus type	GOP	Description
HBV	01810	HBs antigen test
	01932	HBs antigen and HBc antibodies before starting pre-exposure HIV prophylaxis
	32781	Detection of HBsAg
	32614	HBc antibodies
	32615	HBc IgM antibodies
	32616	HBe antibodies
	32782	Detection of HBeAg
	32823	Hepatitis B virus DNA or hepatitis C virus RNA, quantitative
HDV	32619	HDV antibodies
	32620	HDV IgM antibodies

HBsAg: Hepatitis B surface antigen; HBc: Hepatitis B core antigen; HIV: Human Immunodeficiency Virus; IgM: Immunoglobulin M; HBeAg: Hepatitis B e antigen; DNA: Deoxyribonucleic Acid; RNA: Ribonucleic Acid; HDV: Hepatitis D virus

* All GOPs are valid for the whole analysis period except for 01932 (valid from 2019 onwards)

**Table 2:** Combination of GOPs for different groups

Virus type	Group	Combination of GOPs
HBV	Persons tested with ≥ 1 marker	(01810 **OR** 01932 **OR** 32781 **OR** 32614)
	Persons tested with ≥ 2 markers	(01810 **OR** 01932 **OR** 32781 **OR** 32614) **AND** (32614 **OR** 32615 **OR** 32616 **OR** 32782 **OR** 32823)
HDV	Persons tested for HDV	(32619 **OR** 32620)
	Persons tested for HDV and ≥ 2 HBV markers in the same quarter	(32619 **OR** 32620) **AND** (01810 **OR** 01932 **OR** 32781 **OR** 32614) **AND** (32614 **OR** 32615 **OR** 32616 **OR** 32782 **OR** 32823)

*32614 used for persons tested with ≥ 2 HBV markers if the other marker was HBsAg (01810; 01932; 32781)

#### Persons tested for HDV

We identified persons with at least one HDV antibody or HDV IgM antibody test within the respective analysis year. As reimbursement for molecular HDV testing (HDV DNA/RNA) was only introduced in 2021, these markers could not be included in our analysis covering 2016–2021. Additionally, we identified persons tested for HDV with ≥ 2 HBV markers in the same quarter.

#### Prevalent HBV infections

Patients were classified as having prevalent HBV infection in a given analysis year if they met the case definition (≥ 1 inpatient diagnosis or ≥ 2 confirmed outpatient diagnoses recorded in different quarters (M2Q criterion) within a 12-month period). For outpatient diagnoses, the 12-month assessment window was applied on a rolling basis across four quarters. If the required outpatient diagnoses spanned two calendar years, cases were assigned to the year of the first qualifying diagnosis (index year). Thus, prevalence was assessed on a yearly basis, counting all patients assigned to the respective analysis year. Diagnoses were coded in accordance with the ICD codes for HBV: B16.0, B16.1, B16.2, B16.9, B17.0, B18.0, B18.1. ICD codes for HBV comprise acute and chronic infection with and without HDV coinfection. We did not stratify by acute/chronic status, as outpatient HBV infections were considered if ≥ 2 ICD codes were present, irrespective of disease stage. For description of ICD codes used, see supplementary table S1.

#### Prevalent HDV infections among HBV-positives

We identified HDV prevalent infections using a similar approach to the HBV definition, based on the following ICD codes: B16.0, B16.1, B17.0, B18.0 in the respective analysis year. The ICD codes capture both simultaneous infection and superinfection.

#### Incidence of newly detected HBV infections

Newly detected HBV infections were defined as patients meeting the definition of HBV infection (≥ 1 inpatient diagnosis or ≥ 2 confirmed outpatient diagnoses recorded in different quarters (M2Q criterion) within a 12-month period) as the first recorded diagnosis following a 3-year diagnosis-free interval. This interval was applied to increase the likelihood that cases reflected newly identified infections rather than ongoing or previously diagnosed infections reappearing in claims data. The choice of a 3-year diagnosis-free interval was based on literature review [16, 17], expert opinion and sensitivity analyses using alternative intervals, which suggested that a 3-year interval reduces misclassification of prevalent cases as incident cases and allows for the availability of at least two data points within the analysis period. Due to the diagnosis-free interval, incident infections were assessed for 2019 and 2020 only.

### Statistical methods

Data included information on sex, age and age groups (stratified into 0–29, 30–39, 40–49, 50–59, 60–69, 70–79, ≥ 80 years) with regard to data availability. For categorical variables we calculated frequencies and proportions. For continuous variables we calculated mean and standard deviation (SD).

The **number of persons tested** are displayed per 100,000 individuals of the German total population. The **annual proportion of prevalent HBV infections** was calculated by dividing the number of prevalent HBV patients by the total cohort included in the respective analysis year. To calculate the proportion of **annual prevalent HDV infections among HBV-positive persons**, we divided the number of individuals with HDV infection by the number of individuals with prevalent HBV infection in the respective analysis year [17]. **Incidence of newly detected HBV infections** was calculated by dividing the number of newly detected HBV patients by the total at-risk study population (3-year diagnosis-free interval prior to first diagnoses within the analysis period). Incidence of newly detected HBV infections is displayed per 100,000 persons each year with corresponding 95% confidence intervals (95% CI).

The number of patients with annual prevalent and newly detected infections were extrapolated to the total German population by using age- and gender-standardisation. For extrapolation, we applied weights based on official population data from the German Federal Bureau of Statistics (Statistisches Bundesamt, DESTATIS) [18], corresponding to the specific analysis year (2016–2021) to ensure that annual estimates correspond to the population structure of the respective year. This involved calculating weighted averages of stratum-specific rates and assigning age- and sex-specific weights to each individual in the InGef database. The total sum of weighted results corresponds to the estimated number of individuals in the German population. Weighted calculations were consistently applied to all subgroups to ensure representativeness. Analyses were performed using R version 4.3.2.

## Results

### Number of persons tested for HBV and HDV

**Fig. 2:**
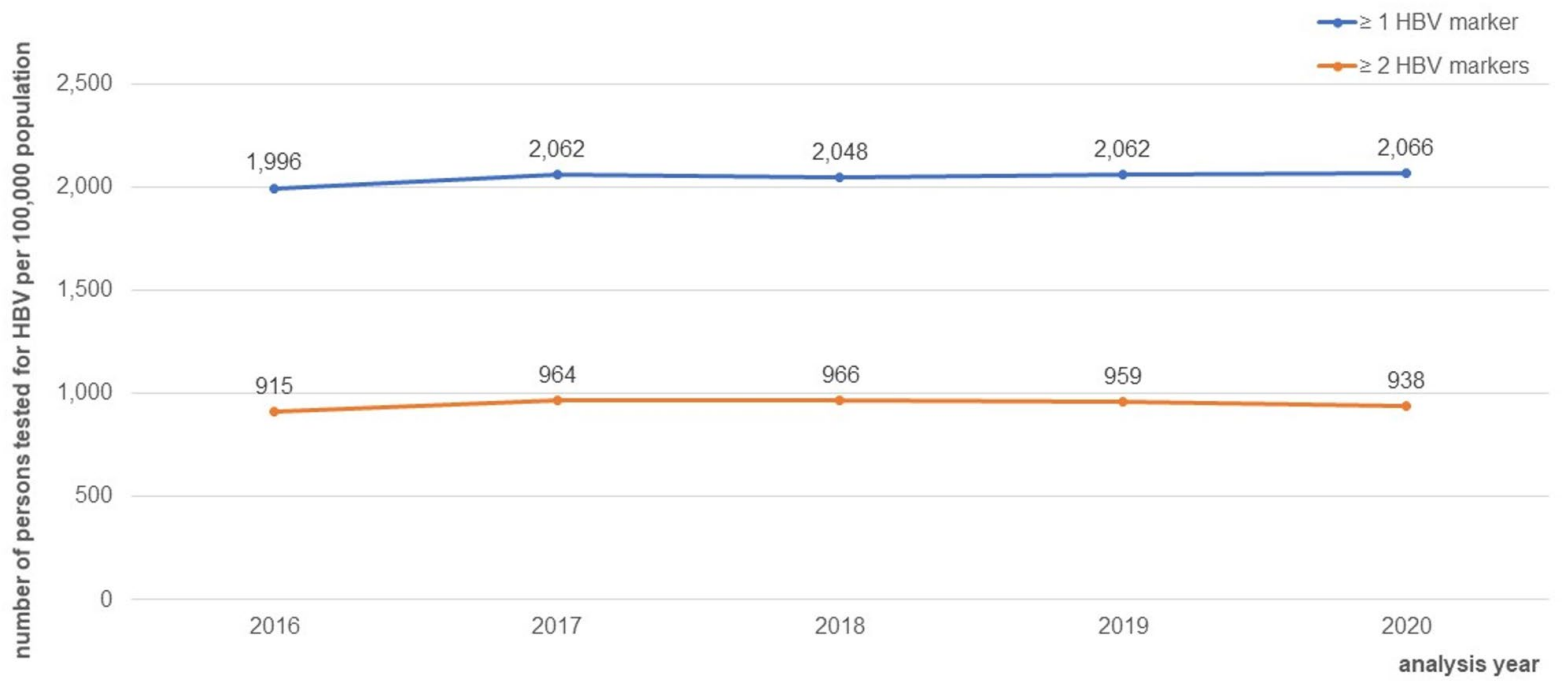
Number of persons tested for HBV per 100,000 of the German total population, persons tested for at least one HBsAg or Anti-HBc test (≥ 1 marker), and persons tested for ≥ 2 markers within the same quarter, regardless to combination: (HBsAg **OR** Anti-HBc) **AND** (DNA **OR** HBeAg **OR** Anti-HBc **OR** Anti-HBc IgM **OR** Anti-HBe), 2016–2020

The number of persons with at least one HBsAg or Anti-HBc test (≥ 1 marker) was 1,996 to 2,066 per 100,000 population between 2016 and 2020. Nearly all (97%) of the persons with ≥ 1 marker were tested for HBsAg only. The number of persons with ≥ 2 markers varied from 915 to 966 per 100,000 population (see Fig. 2).

**Fig. 3:**
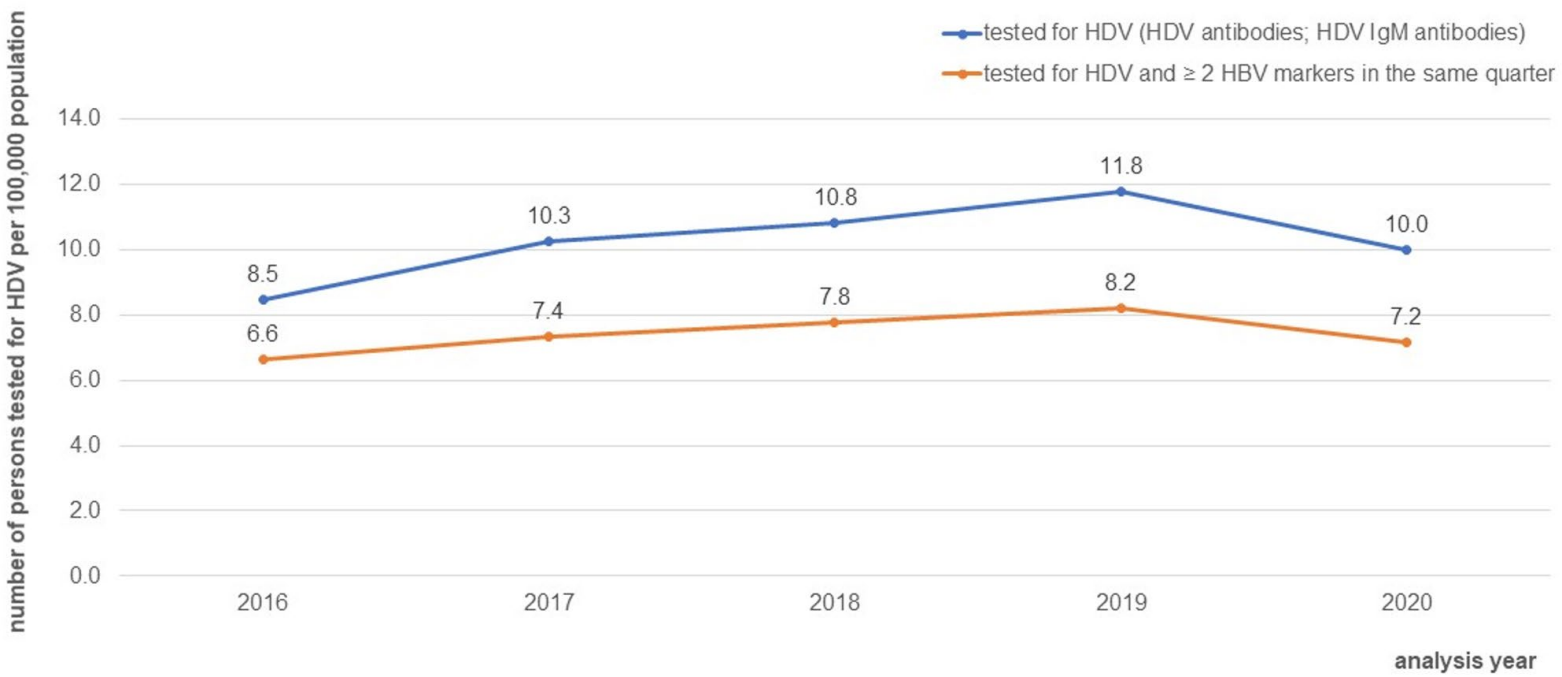
Number of persons tested for HDV and persons tested for HDV and ≥ 2 HBV markers in the same quarter per 100,000 of the German total population, 2016–2020

The number of persons tested for HDV resulted in 8.5 to 11.8 per 100,000 population annually (see Fig. 3). 69.5% to 77.6% of persons tested for HDV were tested for ≥ 2 HBV markers in the same quarter.

### Prevalent HBV infections

**Fig. 4:**
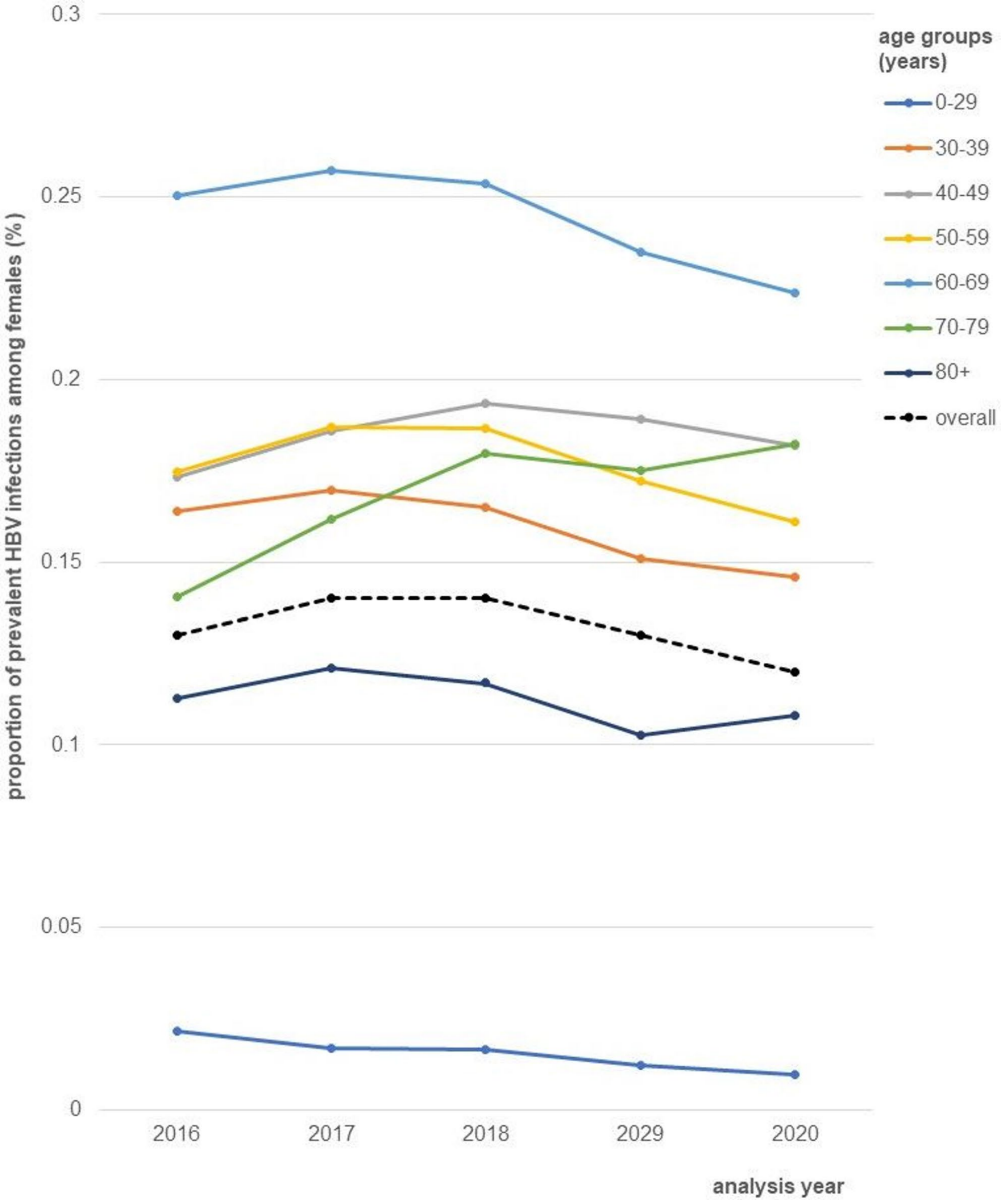
Annual proportion of prevalent HBV infections (%) among females, extrapolated to the German total population, stratified by age groups (years), 2016–2020

**Fig. 5:**
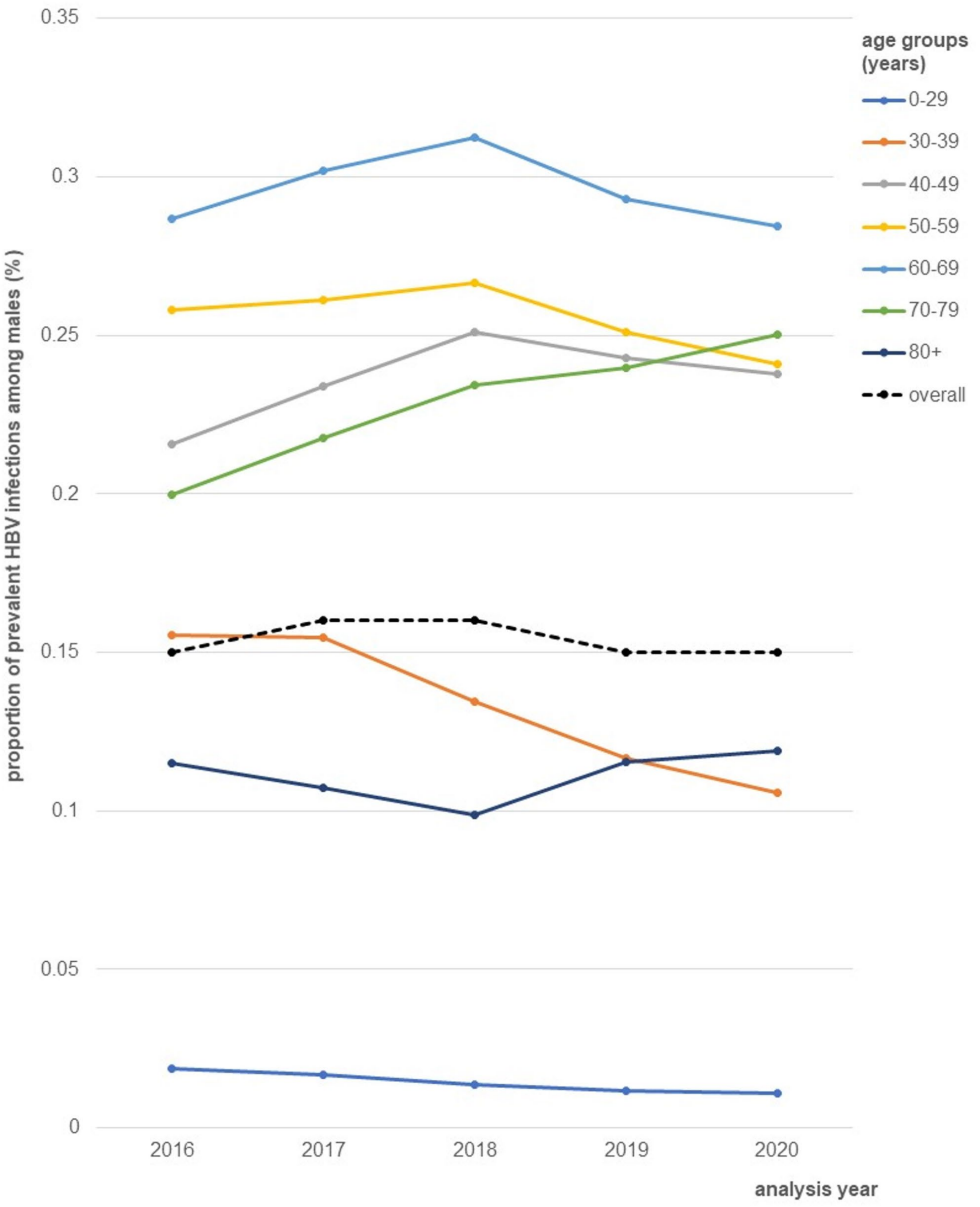
Annual proportion of prevalent HBV infections (%) among males, extrapolated to the German total population, stratified by age groups (years), 2016–2020

The average proportion of HBV prevalent infections was 0.14% with annually variation from 0.14% (95% CI: 0.14; 0.14) to 0.15% (95% CI: 0.15; 0.15) between 2016 and 2020. Detailed information by analysis year and 95% CI are provided in supplementary table S2. The mean age of individuals with prevalent infection varied from 55 to 57 years (SD = 15) and did not differ by sex (supplementary table S4). The annual proportion of prevalent HBV infections by age group stratified by sex is illustrated in Fig. 4 for females and Fig. 5 for males. In both populations, individuals aged 60 to 69 years showed the highest proportion (females: 0.22–0.26%; 2016–2020; males: 0.28–0.31%; 2016–2020). For both sexes, the lowest proportion of prevalent infections was found among individuals aged 0 to 29 years (range: 0.01–0.02%; 2016–2020). The proportion of HBV prevalent infections was slightly higher among males (0.15–0.16%; 2016–2020) than females (0.12–0.14%; 2016–2020).

### Prevalent HDV infections among HBV-positive persons

The annual proportion of prevalent HDV infections among HBV-positive persons varied from 4.5 to 6.4% between 2016 and 2020. The small number of infections in the underlying dataset does not allow any further meaningful stratification according to age or sex.

### Incidence of newly detected HBV infections (3-year diagnosis-free interval)

In 2019, the number of newly detected HBV infections (3-year diagnosis-free interval) among the German total population was 11,268, which corresponds to an incidence of 13.5 per 100,000 (95% CI: 13.3; 13.8). In 2020, the number of individuals dropped to 7,392, resulting in a nationwide incidence of newly detected HBV infections of 8.9 per 100,000 (95% CI: 8.7; 9.1). Detailed information by analysis year and 95% CI are provided in supplementary table S3.

**Fig. 6:**
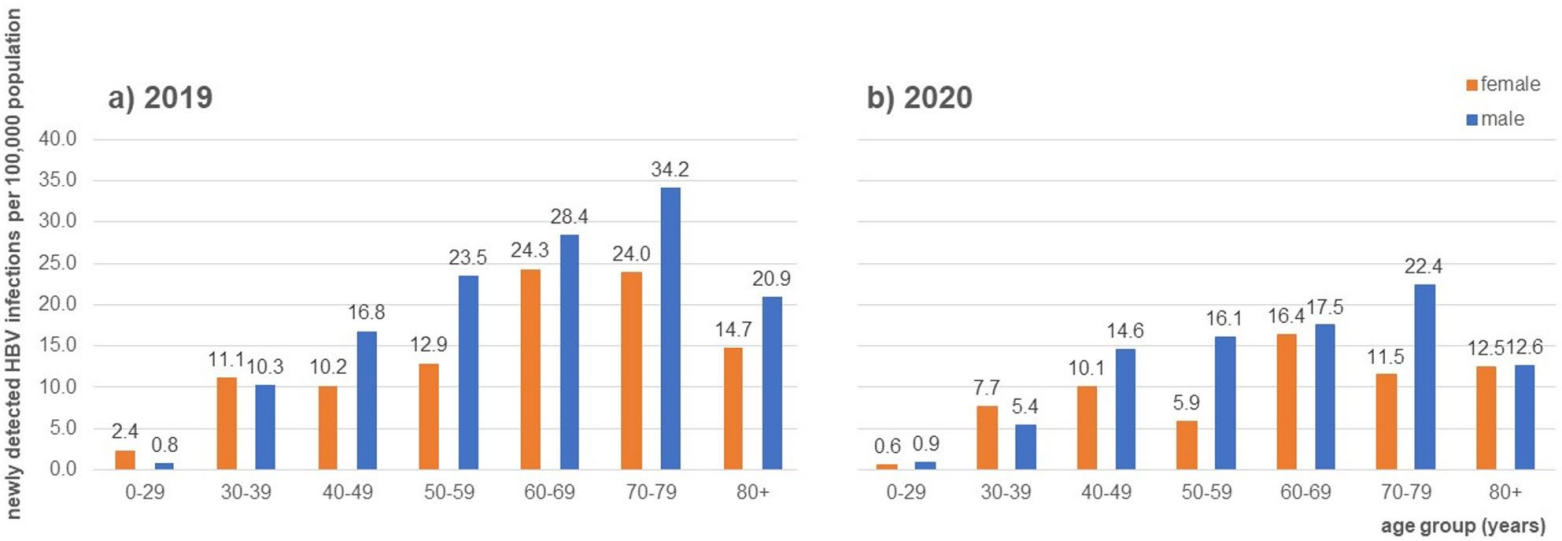
Incidence of newly detected HBV infections (3-year diagnosis-free interval) per 100,000 in 2019 (**a**) and 2020 (**b**), extrapolated to the German total population, stratified by age groups (years) and sex

The mean age of individuals with newly detected infection was 60 years (SD = 16) and did not differ by sex (supplementary table S5). Among males, the incidence of newly detected HBV infections was higher (15.1 per 100,000 in 2019, 10.2 per 100,000 in 2020) than among females (12.0 per 100,000 in 2019, 7.6 per 100,000 in 2020). Among males, the highest proportion of newly detected infections was aged 70 to 79 years (34.2 per 100,000 in 2019, 22.4 per 100,000 in 2020). The highest proportion of newly detected infections among females was seen in the age group of 60 to 69 years (24.3 per 100,000 in 2019, 16.4 per 100,000 in 2020). For both sexes, the lowest number of incident infections was among individuals aged 0 to 29 years (see Fig. 6).

## Discussion

To our knowledge, this is the first study examining claims data as source for generation of monitoring indicators for viral hepatitis in Germany. We derived plausible values for the annual number of persons tested for HBV and HDV as well as prevalent infections and incidence of newly detected HBV infections (3-year diagnosis-free interval). Overall, our findings suggest that claims data can be used for the continuous generation of monitoring indicators and meet several suitability criteria, including the ability to provide plausible and internally consistent trends and to capture testing activities over time. At the same time, specific limitations need to be acknowledged when interpreting the results, which are outlined in detail in the following sections of the discussion.

### Number of persons tested for HBV and HDV

We identified that approximately 2,000 persons per 100,000 population were annually tested for at least one marker (HBsAg or Anti-HBc) and around one half of these persons were tested for 2 or more different HBV markers. We conclude that claims data are able to quantify and monitor testing activities over time. Nonetheless, true testing rates may be underestimated because our analysis includes only tests reimbursed in the outpatient sector among statutorily insured individuals, while testing in hospitals, prisons or uninsured populations is not captured. This limitation aligns with the broader challenge that claims data cannot reflect all testing settings and may underrepresent vulnerable groups.

We compared our findings to data from other countries in the European Union [7]. France reports a higher number of persons tested (approx. 7,000 per 100,000 population), reflecting a broader testing population. Our findings align with data from Malta and Hungary; however, Germany’s testing strategy targets broader vulnerable groups (e.g. men who have sex with men, people who inject drugs, migrants from high-endemic countries), unlike Malta [7]. As stated by the ECDC, survey data has limitations regarding availability and quality of data and specific local or national testing strategies reduce comparability [7]. In the broader European context, these comparisons indicate that testing activities in Germany will need to increase to reach levels observed in other EU countries and to support progress toward elimination goals.

Our data analysis includes data up to the year 2020. Since then, the number of performed tests likely increased from October 2021 onwards after the introduction of one-time HBV and HCV screening for persons from the age of 35 attending a health examination. Since 2022, the number of HBV notification has raised substantially in people aged 35+, pointing towards increased testing activities [19, 20].

The number of persons tested for HDV resulted in 8.5 to 11.8 per 100,000 population between 2016 and 2020. According to German national guidelines [15], persons tested positive for HBV should receive HDV reflex testing. Herta et al. (2025) found that only 22% of HBsAg-positive individuals in German primary care were tested for HDV between 2012 and 2021, leading to the recommendation to include HDV reflex testing in the HBV/HCV screening for adults aged 35+ [21]. Although we did not directly measure reflex testing, HBV incidence (13.5 per 100,000) in 2019 was higher than the number of persons tested for HDV with ≥ 2 HBV markers in the same quarter (8.2 per 100,000), supporting a clear gap in reflex testing. These findings demonstrate that claims data can identify opportunities for public health action.

### Prevalent HBV infections

The average proportion of annual prevalent HBV infections was 0.14% and remained stable over time. This estimate is lower than the 0.3% prevalence reported in nationwide German seroprevalence data (2008–2011) [9]. After excluding vulnerable groups from these data, Kremer-Flach et al. reported 0.15% prevalence [10], that is aligning with our findings. Vulnerable groups, particularly people with a history of migration - who show higher HBV prevalence [22, 23] - are likely underrepresented in the InGef sample due to health insurance structures [24]. Additionally, prevalence might have changed over time. Since claims data reflect only diagnosed infections, undetected infections remain outside the system, making estimate from claims data a proxy for the lowest possible prevalence. Despite this limitation, claims data provide a reliable and longitudinally monitorable baseline for continuous hepatitis monitoring. This baseline can be generated alongside other indicators and offer public health authorities valuable insights regarding changes over time. However, this estimate cannot replace a representative seroprevalence study that would include also undiagnosed and uninsured populations.

The mean age of prevalent infections varied from 55 to 57 years. Several factors may have influenced this finding: the long asymptomatic course of HBV and age-related multimorbidity (especially if the hepatitis infection is causing liver disease). Also, elderly cohorts show a higher infection risk in young ages with regard to historical gaps in infection prevention and control measures. Additionally, due to recommendation of HBV childhood vaccination in 1995, earlier birth cohorts did not benefit from universal childhood immunisation [25]. An US claims study reported comparable mean ages in chronic HBV patients: 51.8 years (commercial/Medicare) and 50.2 years (Medicaid) in 2015, recognizing an increasing trend since 2006 [26]. Similarly, an Asian cohort study of chronic HBV patients found a mean age of 55 years in 2014–2017, also noting a rise since 2000 [27]. These findings indicate that older cohorts may require targeted screening and intervention strategies to address long-standing infections and associated complications.

### Prevalent HDV infections among HBV-positive persons

The proportion of prevalent HDV infections among HBV-positive persons obtained from this study ranged from 4.5% to 6.4% by analysis year. Our results are comparable to an estimated prevalence of HDV/HBV co-infections from 0% to 7.4% in Germany [28], as well as to a global HDV prevalence of 4.5% among HBsAg-positive persons, reported by a systematic review and meta-analysis, that estimated a prevalence of 6.6% in Germany [29].

As vulnerable groups are likely underrepresented in our sample, HDV prevalence may be higher in key populations [21]. Key populations include migrants from high-endemic countries, people who inject drugs and persons coinfected with human immunodeficiency virus or hepatitis C [30, 31]. Additionally, insufficient HDV testing among HBV-positive individuals, should be considered when interpreting the results. Expanding HDV screening among HBV-positive individuals, especially in underserved groups, could improve early detection and support progress toward elimination goals.

### Incidence of newly detected HBV infections (3-year diagnosis-free interval)

In our study, the incidence of newly detected HBV infections (3-year diagnosis-free interval) ranged from 13.5 per 100,000 (2019) to 8.9 per 100,000 (2020). The results are broadly consistent with German surveillance data, reporting 10.7 per 100,000 in 2019 [32] and 8.2 per 100,000 in 2020 [33]. Both sources display acute and chronic HBV infections (and unknown stage in surveillance data), while chronic infections included in surveillance data only since 2019, due to a revised reference definition. A delayed implementation into the reporting system likely causing underestimation in that year.

Both claims and surveillance data show a decline of incidence rates in 2020, probably due to the COVID-19 pandemic and its impact on healthcare services and reduced contacts of the population [34]. In both data sources, a higher number of incident infections among males compared to females could be observed, consistent with findings from other European countries based on The European Surveillance System (TESSy) [35]. Overall, our findings indicate that claims data can reliably track temporal patterns in persons with newly detected HBV infections and their sex distribution, that are consistent with existing surveillance systems.

However, we observed differences in age distribution. While claims data analysis records the highest proportion of newly detected infections among persons aged 60 + years, German surveillance data observes the highest incidence among individuals aged 30–39 years [32, 33]. Data in TESSy from other European countries fits well with German surveillance data, as they record the highest rate of newly diagnosed HBV infections among persons aged 25–44 years [35]. The divergent age distributions indicate that incident infections in older age-groups may be overestimated in claims data, limiting direct comparability with notification data.

Several factors may explain these differences. First, the 3-year diagnosis-free interval used in our study identifies individuals without a documented HBV diagnosis during the preceding three years but does not distinguish between newly acquired and previously undiagnosed chronic infections. Older individuals may therefore be more likely to appear as newly detected cases because long-standing infections are diagnosed only later in life. Consequently, our estimates probably reflect newly diagnosed HBV infections rather than true incident infections. Second, differences in healthcare utilisation may influence case detection. Older individuals generally have more frequent contact with healthcare services and therefore more opportunities for HBV testing and diagnostic coding. In contrast, younger individuals may be underrepresented due to lower healthcare engagement or fewer diagnostic encounters outside targeted screening programmes. The introduction of one-time HBV and HCV screening for individuals aged 35 years and older has likely improved case detection in recent years [19, 20]. In addition, surveillance data face similar challenges. Due to several changes in case definitions and reporting practices, surveillance systems might also capture previously diagnosed infections that were not reported earlier or whose records were no longer available [19]. Furthermore, both surveillance and claims data are likely to underestimate the true number of newly detected infections because asymptomatic or mild infections often remain undiagnosed [25].

Overall, these results highlight that a substantial number of infections likely remain undiagnosed and point to the need for targeted testing strategies that ensure timely diagnosis across all age groups.

### Ability of claims data to capture WHO indicators

Based on the findings and limitations discussed above, we assessed the suitability of claims data for reporting WHO hepatitis indicators. For indicator A.5 (“*Hepatitis B testing*”), claims data provide a robust basis, as reimbursed outpatient testing allows capturing trends over time. As outlined above, testing performed in inpatient settings and among uninsured or hard-to-reach populations is not included, which may lead to an underestimation of overall testing coverage.

Although claims data show internally consistent prevalent infection trends, their ability to reliably distinguish between acute and chronic infections remains uncertain, which limits full alignment with WHO indicator definitions. As we did not stratify by disease stage, we cannot precisely report WHO indicator C.1a (*“prevalence of chronic HBV infection”).* Nonetheless, since claims data reflect tested and diagnosed individuals, we assume that most prevalent infections correspond to chronic infection and consider our estimate to be a reasonable proxy.

As we assume the majority of prevalent HBV infections representing chronic infections, our results can approximate the WHO indicator A.1 (“*Hepatitis D coinfection among people living with chronic HBV infection”).* As discussed in the respective section, the interpretation of this estimate is constrained by the underrepresentation of vulnerable populations with higher HDV prevalence and by incomplete implementation of HDV reflex testing among HBV-positive individuals. These factors are likely to result in an underestimation of true HDV coinfection rates.

Overall, claims data are particularly suitable for monitoring temporal trends in diagnosed HBV infections. However, their suitability for WHO indicators requiring complete population coverage or disease-stage differentiation remains limited due to structural constraints of claims data.

### Strengths of the study

This study has several strengths. First, it is based on a large, population-based claims database that is age- and sex-representative of the statutory health insurance population in Germany, covering approximately 85% of the total population [11]. This allows for robust estimation of hepatitis-related indicators in a real-world setting over a six-year period. Second, the longitudinal and standardised structure of claims data enables the consistent identification of testing activities and newly detected infections over time, which is not routinely available in existing surveillance systems. Third, the study applies operational definitions for multiple hepatitis B and D indicators, allowing for reproducibility and potential transferability of the approach to other healthcare systems using similar claims data structures.

### Limitations of the study

Our results are limited to individuals with statutory health insurance, who have access to healthcare services. Consequently, persons with private health insurance or uninsured persons, including vulnerable populations such as undocumented migrants and homeless people, remained unconsidered. Additionally, the clientele of different German statutory health insurances is historically grown and may differ in social and behavioral factors. Therefore, vulnerable groups with higher prevalence (especially people with a history of migration) may be underrepresented within our analysis sample.

Due to missing stratification for acute and chronic status, we could only approximate WHO indicators C.1a (“prevalence of chronic HBV infection”) and A.1 (“Hepatitis D coinfection among people living with chronic HBV infection”). As claims data do not include laboratory results or information on the reasons for testing, no reliable conclusions can be drawn from the data regarding the causes of changes or differences in testing rates.

Finally, as with all analyses based on claims data, diagnosis codes are collected primarily for reimbursement purposes. Therefore, coding inaccuracies or incomplete documentation cannot be excluded. Although case definitions in this study were designed to increase diagnostic validity (e.g., requiring inpatient diagnoses or repeated outpatient diagnoses using the M2Q criterion), some degree of misclassification may persist. Together, these limitations may lead to both under- or overestimation of the reported outcomes.

## Conclusions

We demonstrate that claims data are a suitable complementary source for generation of monitoring indicators in Germany. Using claims data, we were able to estimate the number of persons tested for HBV and HDV, prevalent and newly detected HBV infections as well as prevalent HDV infections among HBV-positive persons. Claims-derived estimates were generally consistent with existing national data sources and showed plausible trends over time, indicating that claims data can serve as a practical and complementary tool for continuous hepatitis monitoring in Germany, in support of WHO elimination goals. Furthermore, by using claims data, we were able to address a data gap in HBV and HDV testing coverage.

We found that testing coverage in Germany remains limited compared to other EU countries, that the proportion of HBV prevalent infections is stable and the developed indicator can be used as a proxy for the lowest possible prevalence estimate. HDV coinfection is measurable but likely underestimated. The incidence of newly detected HBV infections suggests that a substantial proportion of infections remain undiagnosed or detected late. These patterns underline the importance of strengthening targeted HBV and HDV testing strategies and ensuring earlier detection across all age groups to reduce missed opportunities for care and prevention.

The methodological feasibility demonstrated here may be transferable to other countries and may allow for the efficient and standardised generation of hepatitis monitoring indicators across different contexts.

Analyses should be repeated using the dataset from the new Health Data Lab (Forschungsdatenzentrum, FDZ), which includes all individuals covered by statutory health insurance in Germany, with the aim of routinely generating monitoring indicators. Further research should more granularly assess disease stage -e.g. by combining ICD codes with treatment data- to determine the proportion of persons living with chronic HBV receiving treatment, as well as other indicators from the cascade of care.

## Supplementary Information

Below is the link to the electronic supplementary material.

Supplementary Material 1

## Data Availability

All data generated or analysed during this study are included in this published article and its supplementary information files.

## Abbreviations

EBM: Einheitlicher Bewertungsmaßstab (eng: doctor’s fee scale)
ECDC: European Centre for Disease Prevention and Control
FDZ: Forschungsdatenzentrum (eng: Health Data Lab)
GDPR: General Data Protection Regulation
GOP: Gebührenordnungsposition (eng: fee schedule items)
HBV: Hepatitis B virus
HCC: Hepatocellular carcinoma
HDV: Hepatitis D virus
ICD: International Classification of Diseases
InGef: Institut für angewandte Gesundheitsforschung Berlin (eng: Institute for Applied Health Research Berlin)
TESSy: The European Surveillance System
WHO: World Health Organization
