# Supplementary material for: Generating hepatitis B and D monitoring indicators in Germany using claims data: number of persons tested, incident and prevalent infections, 2016–2021
Source: BMC Infect Dis. 2026 Jun 2;26:1087. doi: 10.1186/s12879-026-13706-8 (PMC13238104; doi:10.1186/s12879-026-13706-8)
Supplement: Supplementary file 1 — Supplementary Material 1 [file 12879_2026_13706_MOESM1_ESM.docx]

# **SUPPLEMENT**

# **Generating hepatitis B and D monitoring indicators in Germany using claims data: number of persons tested, incident and prevalent infections, 2016-2021**

Branke, L.^1,2^, Hofmann, A.^1^, Stepanovich-Falke, A. ^1,2^, Alibone, M.^3^, Bremer, V.^1^, Zimmermann, R.^1^, Dudareva, S^1,4^.

1 Department of Infectious Disease Epidemiology, Robert Koch Institute, Berlin, Germany

2 Charité – Universitätsmedizin Berlin, corporate member of Freie Universität Berlin and Humboldt-Universität zu Berlin

3 InGef - Institute for Applied Health Research Berlin GmbH [Institut für angewandte Gesundheitsforschung Berlin GmbH], Germany

4 Institute of Public Health, Riga Stradins University, Riga, Latvia

# **Table S1: Description of ICD codes used in definitions**

| **Virus type** | **ICD-Codes** | **Description** |
| --- | --- | --- |
| HBV/  HDV  Coinfection | B16.0 | Acute viral hepatitis B with delta virus (co-infection) and with hepatic coma |
|  | B16.1 | Acute viral hepatitis B with delta virus (co-infection) without hepatic coma |
|  | B17.0 | Acute delta virus (super) infection of a hepatitis B (virus) carrier |
|  | B18.0 | Chronic viral hepatitis B with delta virus |
| HBV | B16.2 | Acute viral hepatitis B without delta virus with hepatic coma |
|  | B16.9 | Acute viral hepatitis B without delta virus and without hepatic coma |
|  | B18.1 | Chronic viral hepatitis B without delta virus |

# **Table S2: Prevalent HBV infections among the German total population per analysis year (stratified by sex and age groups)**

| **Character-istics** | **2016** | | **2017** | | **2018** | | **2019** | | **2020** | |
| --- | --- | --- | --- | --- | --- | --- | --- | --- | --- | --- |
|  | **Prevalence (%)** | **95% CI** | **Prevalence (%)** | **95% CI** | **Prevalence (%)** | **95% CI** | **Prevalence (%)** | **95% CI** | **Prevalence (%)** | **95% CI** |
| **OVERALL** | | | | | | | | | | |
| **Total** | 0.142 | 0.141-0.143 | 0.148 | 0.147-0.149 | 0.150 | 0.149- 0.150 | 0.141 | 0.140- 0.142 | 0.137 | 0.136- 0.138 |
| Age group in years | | | | | | | | | | |
| 0-29 | 0.020 | 0.020-0.021 | 0.017 | 0.016-0.017 | 0.015 | 0.015-0.016 | 0.012 | 0.011-0.012 | 0.010 | 0.010-0.011 |
| 30-39 | 0.160 | 0.157-0.162 | 0.162 | 0.160-0.165 | 0.149 | 0.147-0.152 | 0.133 | 0.131-0.136 | 0.125 | 0.123-0.127 |
| 40-49 | 0.195 | 0.192-0.197 | 0.210 | 0.207-0.213 | 0.222 | 0.220-0.225 | 0.216 | 0.213-0.219 | 0.210 | 0.207-0.213 |
| 50-59 | 0.216 | 0.214-0.219 | 0.224 | 0.222-0.227 | 0.227 | 0.224-0.229 | 0.212 | 0.209-0.214 | 0.201 | 0.199-0.203 |
| 60-69 | 0.268 | 0.265-0.271 | 0.279 | 0.276-0.282 | 0.282 | 0.279-0.285 | 0.263 | 0.260-0.266 | 0.253 | 0.250-0.256 |
| 70-79 | 0.167 | 0.165-0.170 | 0.187 | 0.184-0.190 | 0.205 | 0.201-0.208 | 0.205 | 0.202-0.208 | 0.213 | 0.210-0.217 |
| ≥ 80 | 0.114 | 0.111-0.117 | 0.116 | 0.113-0.119 | 0.110 | 0.107-0.113 | 0.108 | 0.105-0.110 | 0.112 | 0.110-0.115 |
| **FEMALE** | | | | | | | | | | |
| **Total** | 0.130 | 0.129-0.131 | 0.137 | 0.136-0.138 | 0.138 | 0.137-0.139 | 0.128 | 0.127-0.129 | 0.124 | 0.123-0.125 |
| Age group in years | | | | | | | | | | |
| 0-29 | 0.022 | 0.021-0.022 | 0.017 | 0.016-0.018 | 0.016 | 0.016-0.017 | 0.012 | 0.012-0.013 | 0.010 | 0.009-0.010 |
| 30-39 | 0.164 | 0.160-0.167 | 0.170 | 0.166-0.173 | 0.165 | 0.162-0.169 | 0.151 | 0.148-0.154 | 0.146 | 0.143-0.149 |
| 40-49 | 0.173 | 0.170-0.177 | 0.186 | 0.182-0.190 | 0.193 | 0.190-0.197 | 0.189 | 0.186-0.193 | 0.182 | 0.178-0.186 |
| 50-59 | 0.175 | 0.171-0.178 | 0.187 | 0.184-0.190 | 0.186 | 0.183-0.190 | 0.172 | 0.169-0.175 | 0.161 | 0.158-0.164 |
| 60-69 | 0.251 | 0.246-0.255 | 0.257 | 0.253-0.262 | 0.254 | 0.249-0.258 | 0.235 | 0.231-0.239 | 0.224 | 0.220-0.228 |
| 70-79 | 0.140 | 0.137-0.144 | 0.162 | 0.158-0.166 | 0.180 | 0.176-0.184 | 0.175 | 0.171-0.179 | 0.182 | 0.178-0.186 |
| ≥ 80 | 0.113 | 0.109-0.117 | 0.121 | 0.117-0.125 | 0.117 | 0.113-0.121 | 0.103 | 0.099-0.106 | 0.108 | 0.105-0.111 |
| **MALE** | | | | | | | | | | |
| **Total** | 0.154 | 0.153-0.155 | 0.160 | 0.158-0.161 | 0.161 | 0.160-0.163 | 0.154 | 0.152-0.155 | 0.150 | 0.149-0.151 |
| Age group in years | | | | | | | | | | |
| 0-29 | 0.019 | 0.018-0.019 | 0.017 | 0.016-0.017 | 0.014 | 0.013-0.014 | 0.012 | 0.011-0.012 | 0.011 | 0.011-0.012 |
| 30-39 | 0.155 | 0.152-0.159 | 0.155 | 0.152-0.158 | 0.134 | 0.131-0.138 | 0.117 | 0.114-0.120 | 0.106 | 0.103-0.108 |
| 40-49 | 0.216 | 0.212-0.220 | 0.234 | 0.230-0.238 | 0.251 | 0.247-0.255 | 0.243 | 0.238-0.247 | 0.238 | 0.234-0.242 |
| 50-59 | 0.258 | 0.254-0.262 | 0.261 | 0.257-0.265 | 0.267 | 0.263-0.271 | 0.251 | 0.247-0.255 | 0.241 | 0.237-0.245 |
| 60-69 | 0.287 | 0.282-0.291 | 0.302 | 0.297-0.307 | 0.312 | 0.308-0.317 | 0.293 | 0.288-0.298 | 0.284 | 0.280-0.289 |
| 70-79 | 0.200 | 0.195-0.204 | 0.218 | 0.213-0.223 | 0.234 | 0.229-0.239 | 0.240 | 0.235-0.245 | 0.250 | 0.245-0.256 |
| ≥ 80 | 0.115 | 0.110-0.120 | 0.107 | 0.103-0.112 | 0.099 | 0.094-0.103 | 0.116 | 0.111-0.120 | 0.119 | 0.115-0.124 |

CI: confidence interval

# **Table S3: HBV incidence of newly detected infections (3-year diagnosis-free interval) among the German total population per analysis year (stratified by sex and age groups)**

| **Characteristics** | **2019** | | **2020** | |
| --- | --- | --- | --- | --- |
|  | **Incidence per 100.000** | **95% CI** | **Incidence per 100.000** | **95% CI** |
| **OVERALL** | | | | |
| **Total** | 13.549 | 13.301-13.801 | 8.889 | 8.689-9.094 |
| Age group in years | | | | |
| 0-29 | 1.567 | 1.420-1.730 | 0.741 | 0.642-0.857 |
| 30-39 | 10.691 | 10.091-11.326 | 6.512 | 6.050-7.010 |
| 40-49 | 13.523 | 12.828-14.257 | 12.353 | 11.685-13.058 |
| 50-59 | 18.219 | 17.512-18.955 | 11.026 | 10.476-11.605 |
| 60-69 | 26.307 | 25.344-27.306 | 16.954 | 16.192-17.752 |
| 70-79 | 28.634 | 27.452-29.866 | 16.501 | 15.603-17.450 |
| ≥ 80 | 17.092 | 16.050-18.201 | 12.566 | 11.697-13.501 |
| **FEMALE** | | | | |
| **Total** | 12.030 | 11.703-12.365 | 7.582 | 7.323-7.849 |
| Age group in years | | | | |
| 0-29 | 2.369 | 2.110-2.660 | 0.567 | 0.448-0.719 |
| 30-39 | 11.116 | 10.252-12.053 | 7.651 | 6.943-8.433 |
| 40-49 | 10.193 | 9.350-11.111 | 10.053 | 9.213-10.970 |
| 50-59 | 12.908 | 12.076-13.798 | 5.871 | 5.316-6.485 |
| 60-69 | 24.304 | 23.026-25.653 | 16.404 | 15.370-17.508 |
| 70-79 | 23.956 | 22.504-25.502 | 11.512 | 10.511-12.608 |
| ≥ 80 | 14.727 | 13.513-16.051 | 12.537 | 11.440-13.739 |
| **MALE** | | | | |
| **Total** | 15.111 | 14.739-15.491 | 10.232 | 9.928-10.547 |
| Age group in years | | | | |
| 0-29 | 0.819 | 0.677-0.990 | 0.904 | 0.754-1.084 |
| 30-39 | 10.284 | 9.472-11.166 | 5.426 | 4.848-6.073 |
| 40-49 | 16.817 | 15.731-17.978 | 14.632 | 13.615-15.725 |
| 50-59 | 23.482 | 22.354-24.666 | 16.140 | 15.204-17.132 |
| 60-69 | 28.437 | 27.010-29.940 | 17.518 | 16.417-18.693 |
| 70-79 | 34.161 | 32.266-36.167 | 22.405 | 20.870-24.052 |
| ≥ 80 | 20.935 | 19.094-22.953 | 12.614 | 11.239-14.157 |

CI: confidence interval

# **Table S4: Characteristics of individuals with prevalent HBV infection among the German total population per analysis year**

| **Characteristics** | **2016** | **2017** | **2018** | **2019** | **2020** |
| --- | --- | --- | --- | --- | --- |
| **Denominator (N)** | 82,521,653 | 82,792,351 | 83,019,213 | 83,166,711 | 83,155,031 |
| **Total cases (n)** | 117,009 | 122,486 | 124,142 | 117,064 | 113,765 |
| **Sex (n)** | | | | | |
| Female | 54,292 | 57,309 | 58,018 | 54,047 | 52,306 |
| Male | 62,717 | 65,177 | 66,123 | 63,017 | 61,459 |
| **Mean age in years (SD)** | | | | | |
| Total | 54.8 (15.0) | 55.4 (14.8) | 56.0 (14.6) | 56.4 (14.6) | 56.9 (14.8) |
| Female | 55.1 (15.9) | 56.0 (15.7) | 56.3 (15.5) | 56.5 (15.5) | 57.1 (15.7) |
| Male | 54.5 (14.3) | 55.0 (14.0) | 55.7 (13.8) | 56.3 (13.8) | 56.7 (14.0) |

SD: standard deviation

# **Table S5: Characteristics of individuals with newly detected HBV infection (3-year diagnosis-free interval) among the German total population per analysis year**

| **Characteristics** | **2019** | **2020** |
| --- | --- | --- |
| **Denominator (N)** | 83,166,711 | 83,155,031 |
| **Total cases (n)** | 11,268 | 7,392 |
| **Sex (n)** | | |
| Female | 5,068 | 3,194 |
| Male | 6,201 | 4,198 |
| **Mean age in years (SD)** | | |
| Total | 60.0 (15.8) | 60.0 (15.7) |
| Female | 59.9 (17.2) | 60.8 (17.4) |
| Male | 60.0 (14.6) | 59.4 (14.3) |

SD: standard deviation
